# Supplementary material for: Cooperative regulation of PBI1 and MAPKs controls WRKY45 transcription factor in rice immunity
Source: Nat Commun. 2022 May 16;13:2397. doi: 10.1038/s41467-022-30131-y (PMC9110426; doi:10.1038/s41467-022-30131-y)
Supplement: Supplementary file 1 — Supplementary Information [file 41467_2022_30131_MOESM1_ESM.pdf]

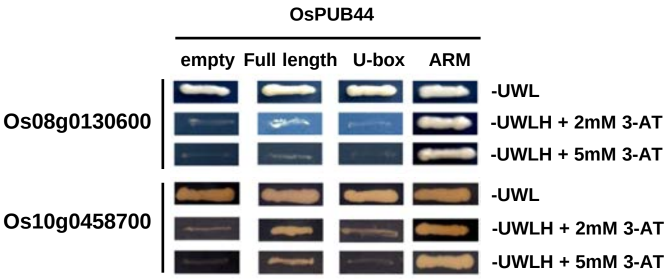

Supplementary Figure 1: Rice factors identified by two hybrid screening.

Two rice proteins were identified by two hybrid screening with the ARM domain of PUB44. Growth of yeast colonies on –ULWH plates with 2 mM or 5 mM 3-aminotriazole (3-AT) indicates a positive interaction. The experiment was repeated three times with similar results.

```
PBI1  MAAEAWRSRFRERVVEAAERWESVGESLAT-ALTHLKSPMHAGDEEEAAAARTRIQLAMG 59
PBI2  MAAEAWRARFRERVVEAAGRWERVREGLAT-ALAHVTSPMLAADEEAAAAARTRIQLAMG 59
PBI3  MAEEAWRERFRQRVAEVDDLFVEAFELLVDNARIHLEAQMLVGD--AAAAERARIQLAQG 58
PBI4  --------MRTPCEIRSSSRWTRS-----------------------ASGMRARAAISPP 29
                     .    :                          *:. *:*  ::

PBI1  ELVDASRNLASAMSLMKVAELLALHGGSVNPSTHLGEISLLGDQYLAERNAGIKLLEAGK 119
PBI2  QLEDASRDLASAMSLMKAADLLALHGDSVNPSTFLGGIGHLGAQYLAERIAVTKLREAWE 119
PBI3  ALEDASGKLASAMSLMVGAKLLVLRGGSHDPLMPYHDIGHLGDEYAAEKNACAKLRGAER 118
PBI4  RFPSRWR-PGSSLSAATASTLLSVR---------------LLRVNYLAEQSAAIKLSYAES 74
       : .     .*::*    : ** ::               *  :* **: *  **  *

PBI1  DARKAYISVDGCRGNLDAILLLLDHP-RVPCVDDFIE-EELFVAGDNLQGAIGNAKLGTE 177
PBI2  DARDAYTNVEWCRSHLDAILLMLDHP-HLPSVDGLIE-EDRAAADGFLQAAIGRAELGNE 177
PBI3  EAEEACARIGMCSGHLETISLLLDHE-NLPGVNDLIENERLDAAVDDLLAAIGKVESGKK 177
PBI4  DARKAYALVDGCRGHLDAALLLLDHVGRLPDVQGMIN-AERLAAVADLEAAIVAVQRSAE 133
      :*..*   :  * .:*::  *:***  .:* *:.:*:     .*   * .**  .: . :

PBI1  RAVGARQDVSGAN 190
PBI2  RAVDARQDVSGAN 190
PBI3  MANDARLDVG-AN 189
PBI4  MATAARQDVSGAS 146
       *  ** **. *.
```

Supplementary Figure 2: Alignment of amino acid sequences of PBI1 – PBI4.

The alignment was created using ClustalW on the DNA Data Bank of Japan website (http://www.ddbj.nig.ac.jp/).

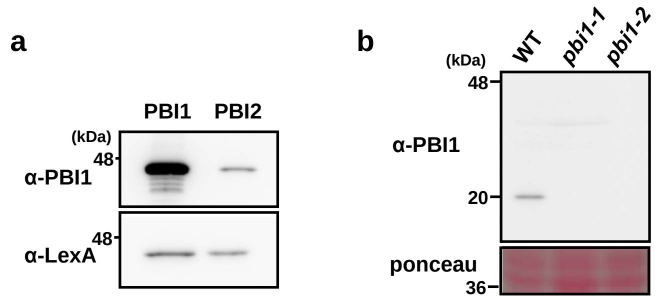

Supplementary Figure 3: Specificity of α-PBI1

a LexA-fused PBI1 and PBI2 proteins were purified from yeast cells and subjected to immunoblots with α-PBI1 or α-LexA. **b** Total proteins were purified from suspension cultured cells of wild type and the *pbi1* mutants, and subjected to immunoblots with α-PBI1. All above experiments were performed three times with similar results.

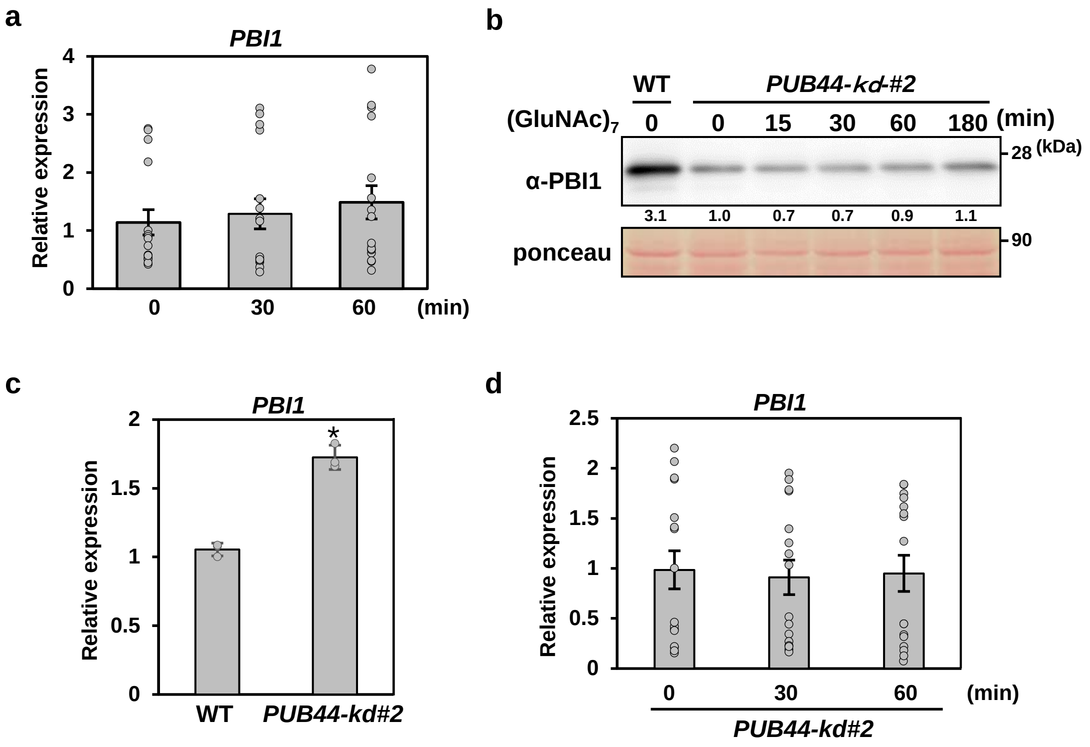

Supplementary Figure 4: The mRNA and protein levels of PBI1 in wild type and the *PUB44-kd* cell line after treatment of chitin.

a *PBI1* transcript levels in wild type cell treated with 2 μg/ml $(GluNAc)_7$ were analyzed using quantitative real-time PCR. Data are means ±SE. n=16 biologically independent replicates. **b** The PBI1 protein levels in the *PUB44-kd#2* cell line were determined by immunoblot analysis with α-PBI1 after treatment with 2 μg/ml $(GluNAc)_7$. The relative abundance of the PBI1 proteins detected is labeled under the blot with α-PBI1. **c** *PBI1* transcript levels in wild type and *PUB44-kd#2* cell line were analyzed using quantitative real-time PCR. Data are means ±SD. n=3 biologically independent replicates. The asterisk indicates statistically significant differences from the WT control by Two-sided Student's t-test ($P < 0.05$). **d** *PBI1* transcript levels in *PUB44-kd#2* cell line treated with 2 μg/ml $(GluNAc)_7$ were analyzed using quantitative real-time PCR. Data are means ±SE. n=16 biologically independent replicates. All above experiments were repeated three times with similar results.

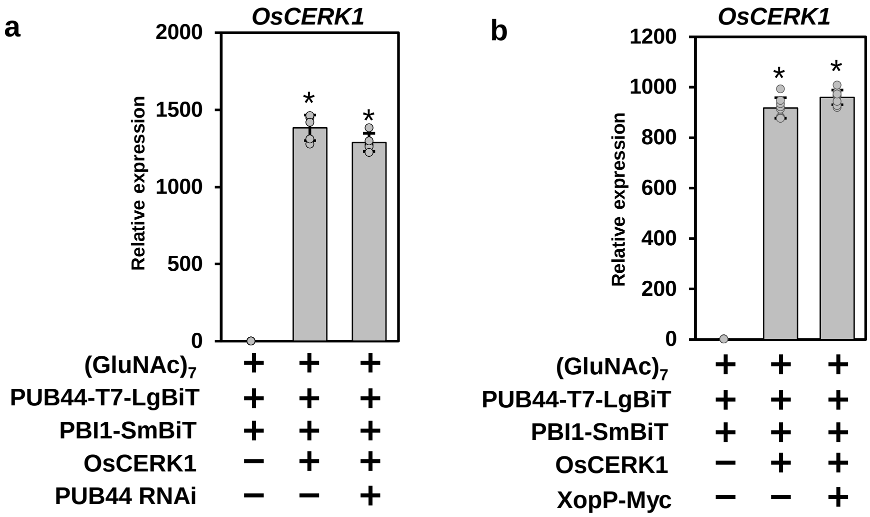

Supplementary Figure 5: The mRNA levels of *OsCERK1* in transient assay

a The mRNA levels of *OsCERK1* in Fig. 2e were analyzed using quantitative real-time PCR. Data are means ±SD. n=5 biologically independent replicates. The asterisk indicates statistically significant differences from the protoplasts that did not carry the *OsCERK1* construct by two-sided Mann-Whitney's U test (P < 0.01). **b** The mRNA levels of *OsCERK1* in Fig. 2i were analyzed using quantitative real-time PCR. Data are means ±SD. n=8 biologically independent replicates.The asterisk indicates statistically significant differences from the protoplasts that did not carry the *OsCERK1* construct by two-sided Mann-Whitney's U test (P < 0.01). All above experiments were performed three times with similar results.

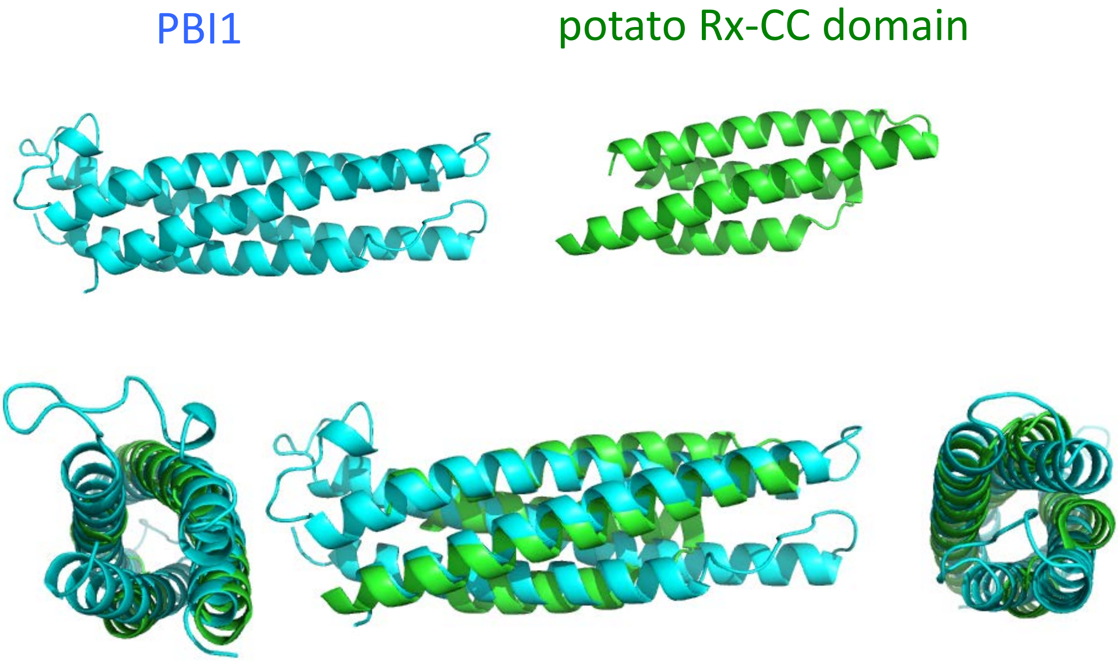

Supplementary Figure 6: Structural alignment of Rx CC domain and PBI1.

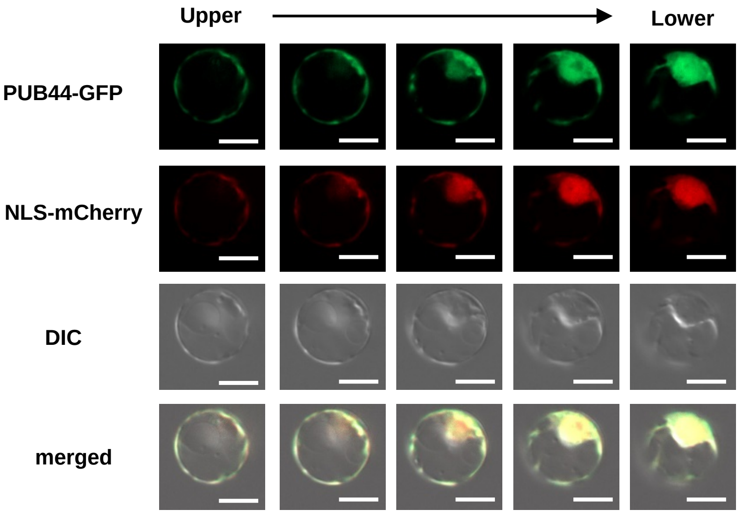

Supplementary Figure 7: Subcellular localization of PUB44-GFP in rice protoplasts.

PUB44-GFP was transiently expressed in rice protoplasts. mCherry with a nuclear localization signal was used as a nuclear localization marker. The fluorescence was observed using a fluorescence microscope, the Axio Imager M2 (Carl Zeiss) with the ApoTome2 system (Carl Zeiss). The section thickness is 1.14 µm. Scale bar=10µm. The experiment was performed three times with similar results.

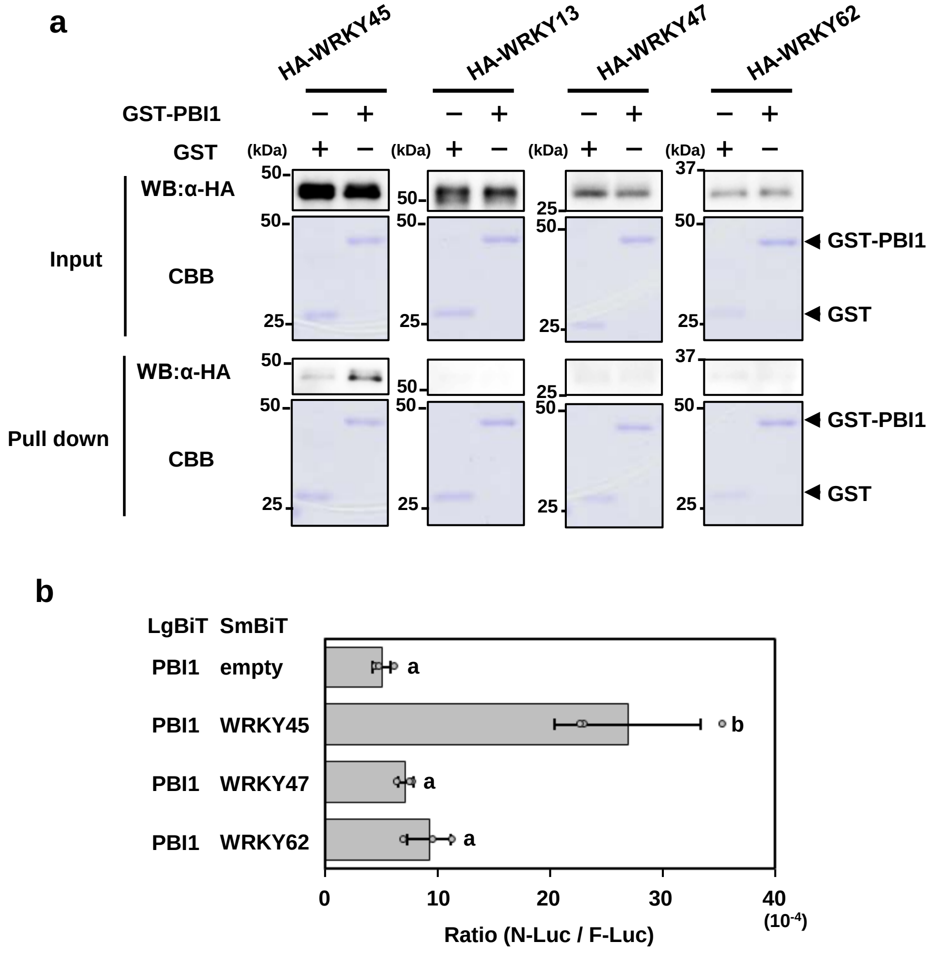

Supplementary Figure 8: PBI1 interacts with WRKY45 but not other WRKYs.

a *In vitro* interaction between PBI1 and WRKYs. GST and GST-fused PBI1 proteins coupled to glutathione Sepharose 4B beads were incubated with HA-tagged WRKY45, HA-WRKY13, HA-WRKY47 and HA-WRKY62. After washing with the buffer containing 0.1 % Triton-X, the proteins were eluted with 40mM glutathione. HA-WRKYs were detected with immunoblotting with α-HA. The GST and GST-fused PBI1 proteins were detected by Coomassie brilliant blue (CBB) staining. **b** The interactions between PBI1 and WRKYs were analyzed using split NanoLuc luciferase complementation assays. PBI1 was fused to LgBiT, and WRKYs were fused to SmBiT. F-Luc was used as an internal control. Rice protoplasts were transfected with the constructs and the interactions were indicated by the N-Luc to F-Luc ratios. Different letters above the data points indicate significant differences ($p < 0.01$, Two-sided Student's *t*-test). Data are means ±SD. n=3 biologically independent replicates. All above experiments were repeated three times with similar results.

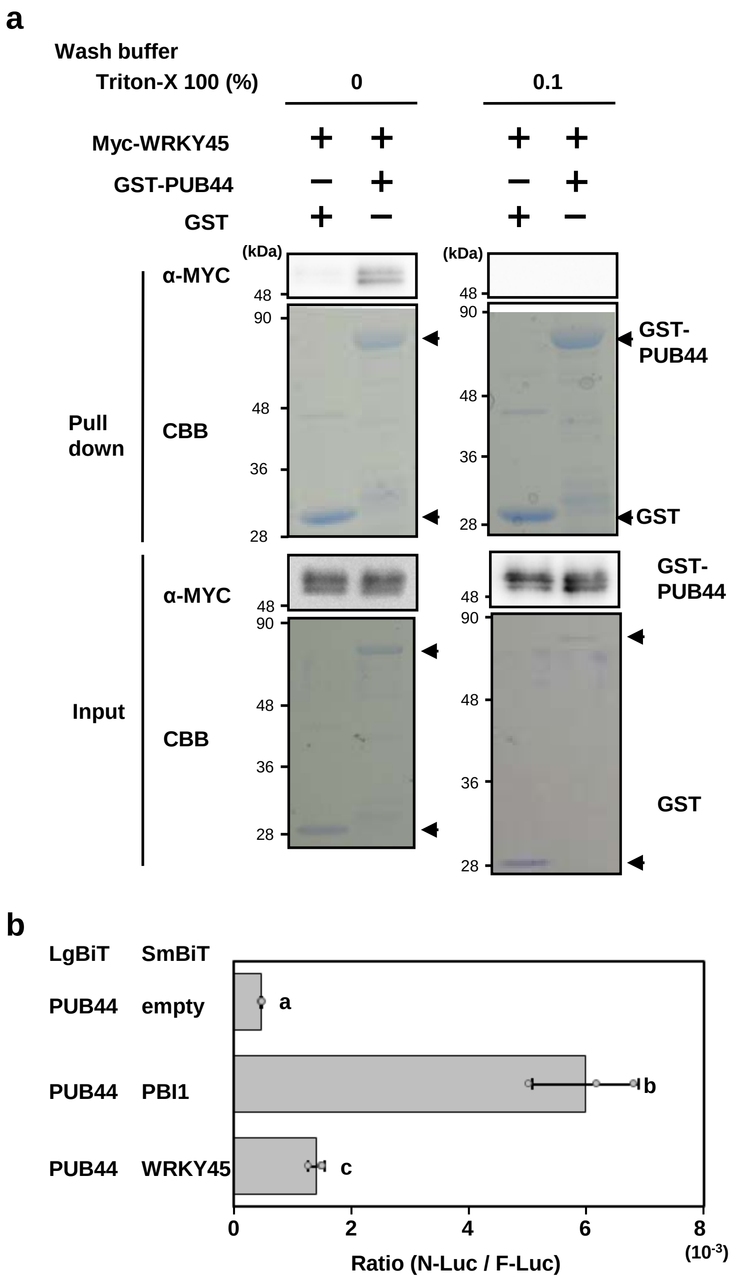

Supplementary Figure 9: PUB44 weakly interacts with WRKY45.

a *In vitro* interaction between PUB44 and WRKY45. GST and GST-fused PUB44 proteins coupled to glutathione Sepharose 4B beads were incubated with Myc-tagged WRKY45 in buffer A (20 mM Tris pH7.5, 50 mM NaCl, 1mM EDTA, 1mM DTT and protease inhibitor) for 2h at 4° C. After washing with buffer A containing 0 % or 0.1 % Triton-X 100, the proteins were eluted with 30mM glutathione. Myc-WRKYs were detected with immunoblotting with α-Myc. The GST and GST-fused PUB44 proteins were detected by Coomassie brilliant blue (CBB) staining of the gel. The interaction between PUB44 and WRKY45 was lost by washing with buffer A containing 0.1% Triton-X 100. **b** The interactions between PUB44 and WRKY45 or PBI1 were analyzed using split NanoLuc luciferase complementation assays. PUB44 was fused to LgBiT, and WRKY45 and PBI1 were fused to SmBiT. F-Luc was used as an internal control. Rice protoplasts were transfected with the constructs and the interactions were indicated by the N-Luc to F-Luc ratios. Data are means ±SD. n=3 biologically independent replicates. Different letters above the data points indicate significant differences ($p < 0.01$, Two-sided Student's *t*-test). All above experiments were performed three times with similar results.

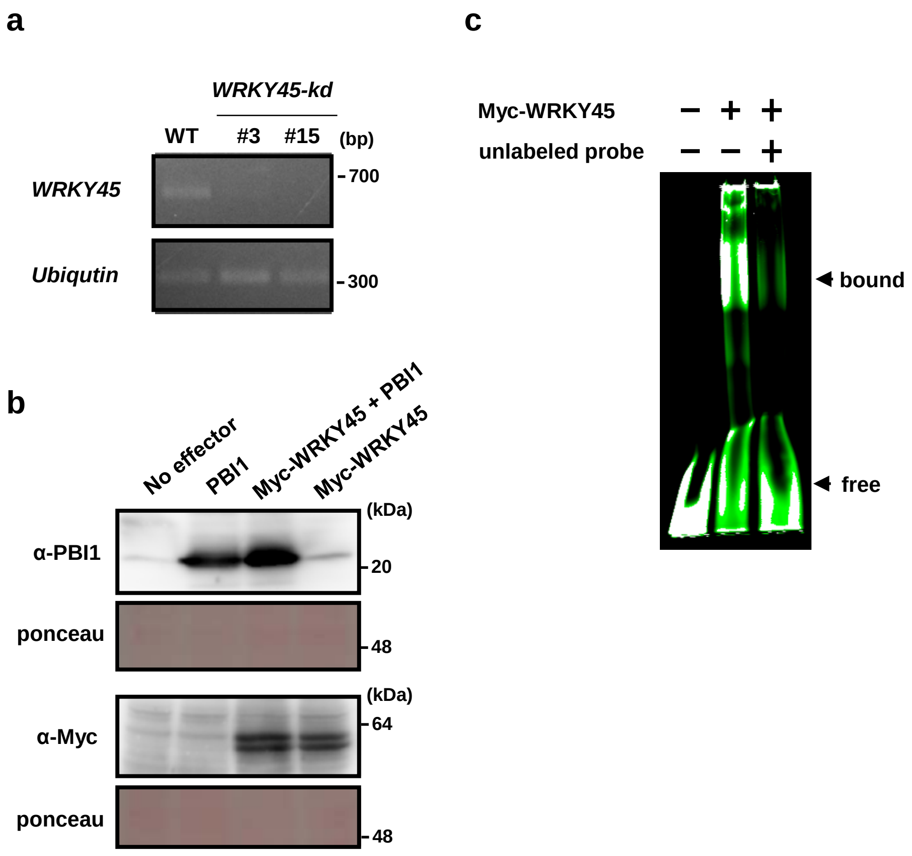

Supplementary Figure 10: PBI1 inhibits WRKY45.

a Transcript levels of *WRKY45* in the suspension cells of the *WRKY45-kd* lines. Semi-quantitative RT-PCR was carried out using specific primers of *WRKY45*. *Ubiquitin* was used as a control. **b** The protein levels of WRKY45-myc and PBI1 in transient Luc assay. Total proteins prepared from the protoplasts transiently expressing WRKY45-myc and PBI1 were subjected to immunoblotting. **c** EMSA was performed using WRKY45 prepared by *in vitro* translation system. Probe containing W-box was labeled by IRDye700. The specificity of W-box binding activity was demonstrated by competition assay using 50-fold excess unlabeled probe. All above experiments were performed three times with similar results.

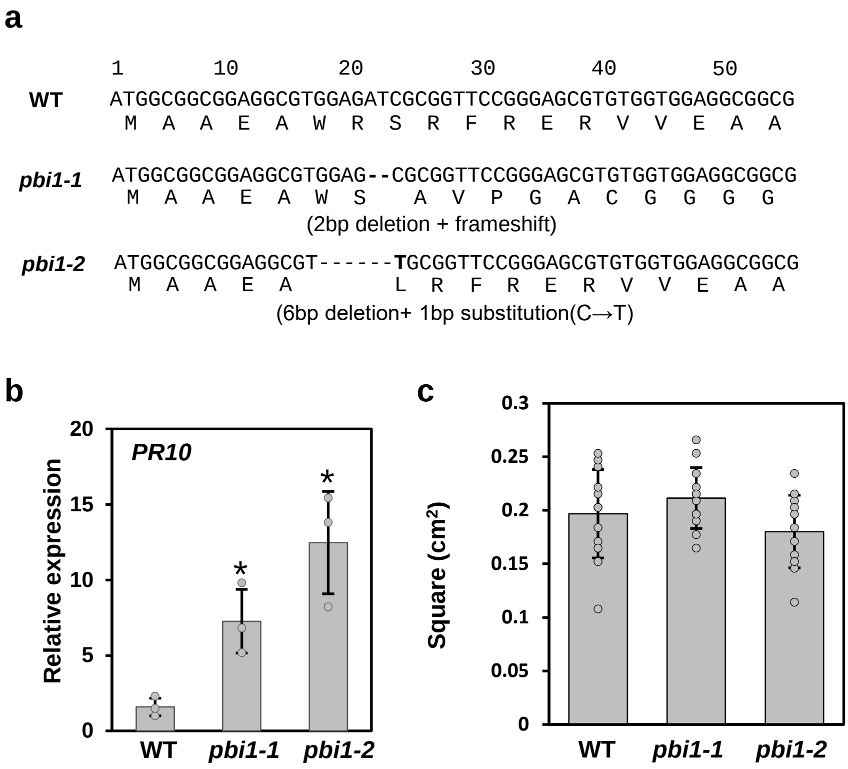

Supplementary Figure 11: Rice blast resistance of the *pbi1* mutants.

a The mutation sites of the *pbi1-1* and *pbi1-2* mutants. **b** Transcript levels of *PR10* in the suspension cells of the *pbi1* mutants. Quantitative real time PCR was carried out using specific primers of *PR10*. *Ubiquitin* was used as a control. Data are means ±SE. n=3 biologically independent replicates. The asterisks indicate statistically significant differences from the WT control by Two-sided Student's t-test ($P < 0.05$). **c** The *pbi1* mutants were infected with the compatible *Magnaporthe oryzae* race ken53-33. Quantitative analysis of disease lesions was performed at 10 days post inoculation. Data are means ±SE. n=12 biologically independent samples. The experiments in (**b**) were repeated three times, and (**c**) twice with similar results.

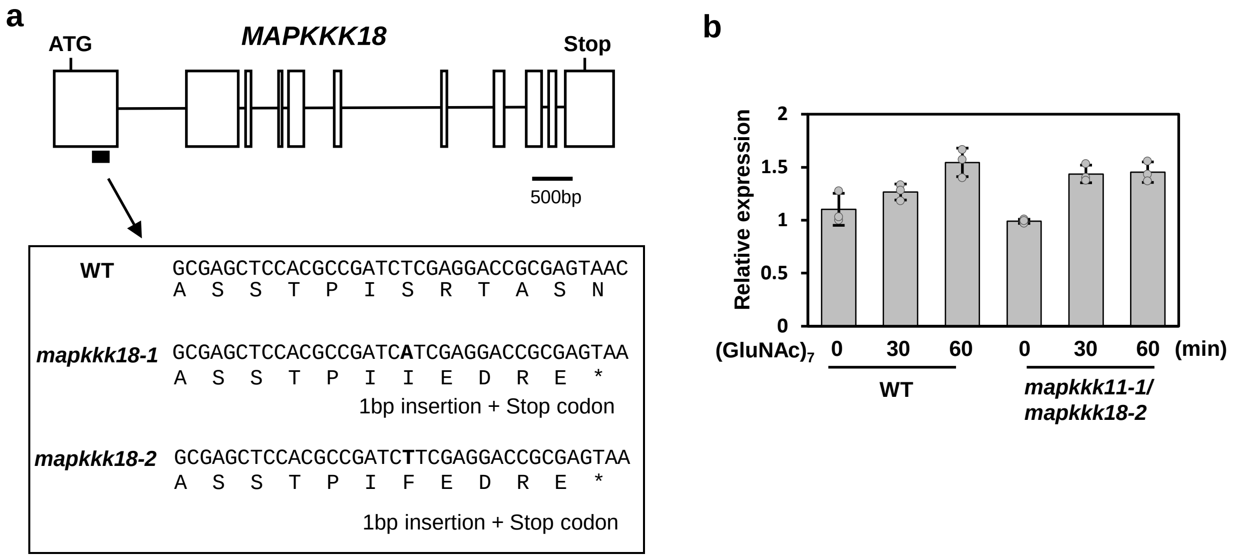

Supplementary Figure 12: *PBI1* expression in the *mapkkk11/18* mutant.

a The mutation sites of *mapkkk18-1* and *mapkkk18-2*. Both mutations contain 1 bp-insertion in the exon1, and create the stop codon. The stop codon was indicated by asterisk **b** *PBI1* transcript levels in the *mapkkk11/18* cells treated with 2 µg/ml $(GluNAc)_7$ were analyzed using quantitative real-time PCR. Data are means ±SD. n=3 biologically independent replicates. Similar results were obtained in three independent experiments.

Supplementary Table1. Data collection, phasing, and refinement statistics of PBI1

	Native-PBI1	SeMet-PBI1
Diffraction source	SPring-8, BL44XU	SPring-8, BL44XU
Wavelength (Å)	0.9000	0.97894
Temperature (K)	100	100
Detector	MX300HE	MX300HE
Crystal-to-detector distance (mm)	250	250
Rotation range per image (°)	0.6	1
Total rotation range (°)	190	185
Exposure time per image (s)	1	1
Space group	$P2_1$	$P2_1$
a, b, c (Å)	89.93, 89.78, 107.09	90.18, 89.76, 107.92
α, β, γ (°)	90, 106.6, 90	90, 106.7, 90
Resolution range (Å)	50.0-1.84 (1.87-1.84)	50.0-2.12 (2.16-2.12)
Total No. of reflections	570139	613163
No. of unique reflections	141131	92035
Completeness (%)	98.0 (97.3)	98.0 (97.3)
Redundancy	4.1 (4.2)	4.1 (4.0)
$\langle I \rangle / \langle \sigma(I) \rangle$	34.3 (3.7)	47.8 (7.0)
R_{merge}*	0.050 (0.418)	0.080 (0.394)
$CC_{1/2}$	0.918	0.963
Refinement		
Overall B factor (Å^2)	29	
$R_{\mathrm{factor}}/R_{\mathrm{free}}$ †	0.193/0.224	
R.m.s. deviations (length, Å/angle, °)	0.011/1.3	
Ramachandran plot ‡		
favored/allowed/outlier (%)	99.4/0.6/0	

Values in parentheses are for highest-resolution shell.

* $R_{merge} = \Sigma_{hkl} \Sigma_i |I_i(hkl) - \langle I(hkl) \rangle| / \Sigma_{hkl} \Sigma_i I_i(hkl)$, where $I_i(hkl)$ is the ith observed intensity of reflection hkl and $\langle I(hkl) \rangle$ is the mean measurement.

† $R_{factor} = \Sigma_{hkl} ||F_{\mathrm{obs}}| - |F_{\mathrm{calc}}|| / \Sigma_{hkl} |F_{\mathrm{obs}}|$, where F_{obs} and F_{calc} are the observed and calculated structure factors, respectively. $R_{free} = \Sigma_{hkl} ||F_{\mathrm{obs}}| - |F_{\mathrm{calc}}|| / \Sigma_{hkl} |F_{\mathrm{obs}}|$ was calculated from a randomly selected 5% subset of reflections that were not used in the refinement.

‡ Ramachandran plot was calculated using MolProbity Validation.

Supplementary Table2. Primers used in the work

cDNA amplification	
PBI1 F	CACCATGGCGGCGGAGGCGTGG
PBI1 R	TCAGTTGGCGCCGGATACATCC
PBI2 F	CACCATGGCGGCGGAGGCGTGGAG
PBI2 R	GAATTCTCAGTTGGCGCCGGATACGTC
PBI3 F	CACCATGGCGGAGGAGGCATGGAG
PBI3 R	TCAGTTCGCGCCGACATCCAG
PBI4 F	CACCATGGCGGCGGAGGTGTGGAAG
PBI4 R	TCACTCTCGGCGTAGCTGAGC
WRKY45 F	CACCATGACGTCATCGATGTC
WRKY45 R	TCAAAAGCTCAAACCCATAATGTCGTC
PUB44-RNAi-F	CACCACCGGAGGATGAGGGAGGCAGT
PUB44-RNAi-R	GCTCGAACGGAAGCAGCATATATA

Real-time PCR	
PBI1 F	TTCTCGTGTTTGCCATTCCA
PBI1 R	TCATCGATCAGACGTCACGAA
WRKY45 F	GACACGGGCCGGGTAAA
WRKY45 R	TTTCTGTACACACGCGTGGAA
WRKY62 F	ACCCCTACCCTAGAGCTTACTTCC
WRKY62 R	GACCTATCCTCCGCACATCTTT
XopA F	ATGAAT TCTTTGAACA CACAATTC
XopA R	TTACTGCATC GATGCGCTGT CGCT
ubiquitin F	CACAAGAAGGTGAAGCTCGC
ubiquitin R	GCCTTCTGGTTGTAGACGTAGG
CERK1 F	CGCGCTGGCTTCCTTCTA
CERK1 R	AAGAGATCCGCCATGTTGGT

Semi qRT-PCR	
ubiquitin F	CACAAGAAGGTGAAGCTCGC
ubiquitin R	GCCTTCTGGTTGTAGACGTAGG
CERK1 F	GCTCGAGTTC TATAGGAGGA GAAAGGC
CERK1 R	GTCTAGACTATCTCCCGGACATTAGGT
PUB44-F	GACTGGATCCGGTACATGGACGCGGCGGTCGCCGGGCAG
PUB44-R	GGCGGCGTGCAGGTGTATGAGCCGGCGCAGGAGCAGGTTGGGGACGAGG
XopP-F	CACCCATATGATGACGCGGTGTGCGCGCAAATTGAATGG
XopP-R	CTCGAGTTCGATATTGTCGACCGG

NanoBit construct	
WRKY45 F-KpnI	GACTGGATCCGGTACATGACGTCATCGATGTCGCC
WRKY45-174R-XhoI	CTGGGTCTAGATATCTTCACTCGCCGATGTAGGTGACCC
WRKY45-174F-KpnI	GACTGGATCCGGTACATGCACACCTGCCGGGACCCGGC
WRKY45 R-XhoI	CTGGGTCTAGATATCTTCAAAAGCTCAAACCCATAATG

HA-tagged protein construct	
1st PCR	
HA-F	CCACCCACCACCACCAATGGAGTACCCATACGACGT
pGADT-R	TGGGGTACAGGTAGTGTTAG
2nd PCR	
SPU	GCGTAGCATTTAGGTGACACT
deSP6E01	GGTGACACTATAGAACTCACCTATCTCCCCAACACCTAATAACATTCAATCACTCT TTCCACTAACCACCTATCTACATCACCACCCACCACCACCAATG
